# Supplementary material for: Connexin26 mediates CO2-dependent regulation of breathing via glial cells of the medulla oblongata
Source: Commun Biol. 2020 Sep 21;3:521. doi: 10.1038/s42003-020-01248-x (PMC7505967; doi:10.1038/s42003-020-01248-x)
Supplement: Supplementary file 1 — Supplementary Information [file 42003_2020_1248_MOESM1_ESM.pdf]

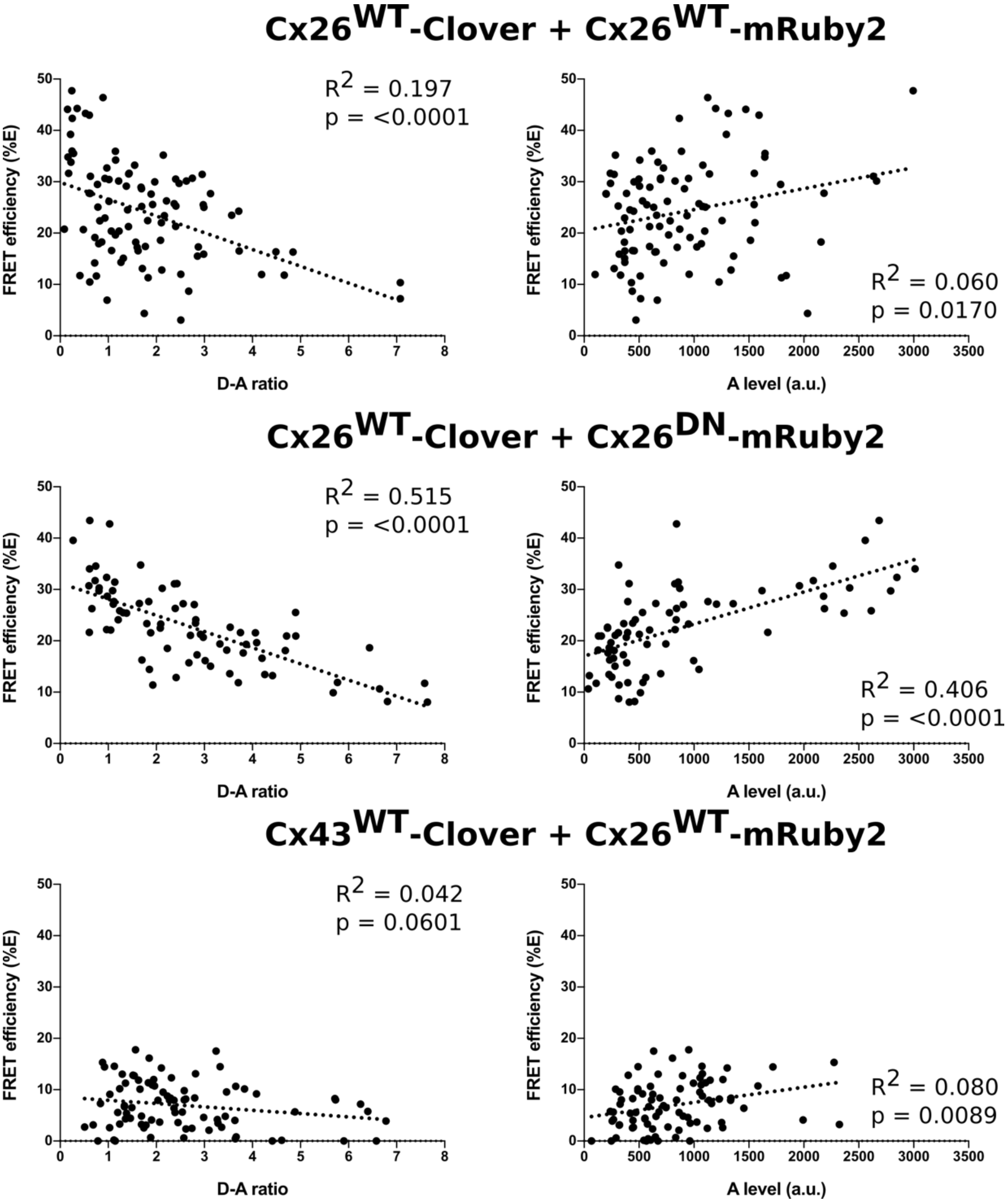

Supplementary Figure 1. The dependence of FRET efficiency (%E) on donor to acceptor (D-A) ratio -see Methods for calculations. A negative correlation between %E and D-A ratio is evidence for coassembly of the subunits into a hexamer (broadly, as the proportion of donor subunits grows relative to the acceptor subunits in the hexamer, the acceptor will saturate and FRET efficiency will decline). A positive correlation between %E and the amount of acceptor (A level), indicates random association between homomeric subunits in the membrane.

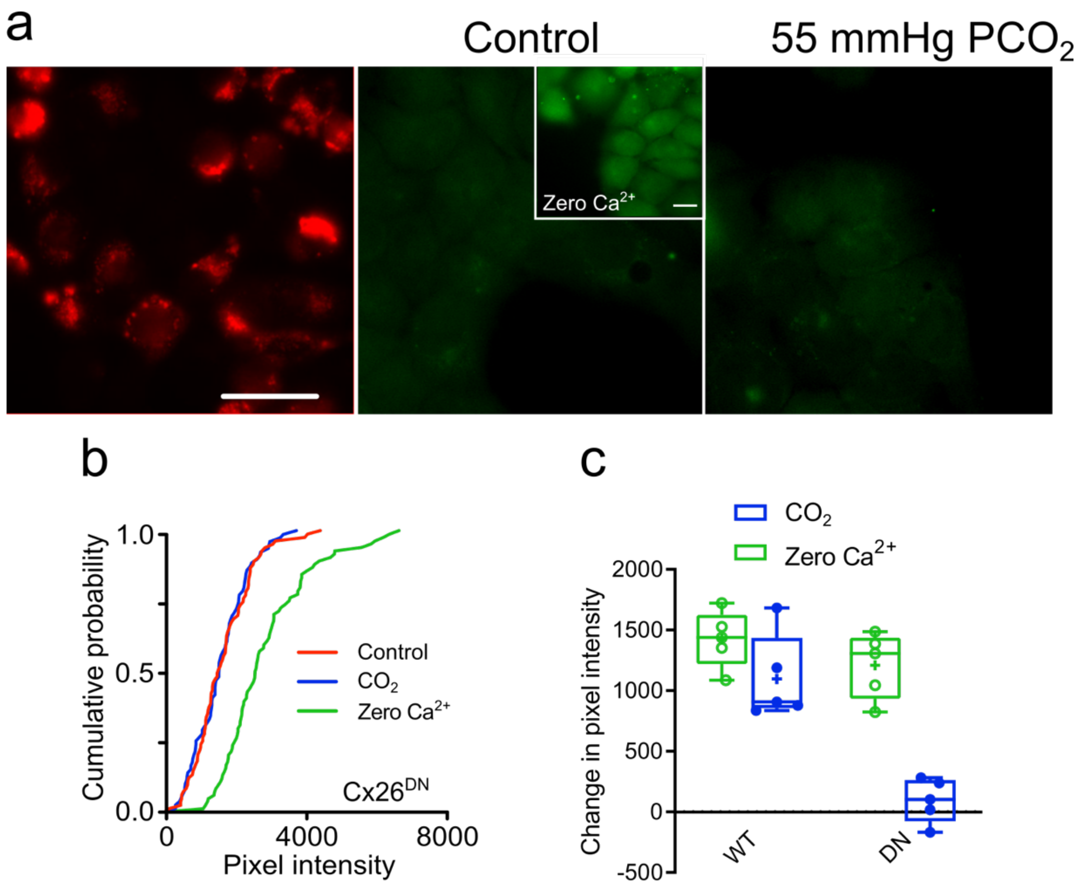

Supplementary Figure 2. $Cx26^{DN}$ is insensitive to CO_2. a) Images of mCherry expression, and dye loading in control and hypercapnic solutions (scale bar 40 µm). The inset demonstrates dye loading in response to zero Ca^{2+} solutions (scale bare 20 µm). This will open hemichannels, and is a positive control to demonstrate the existence of function hemichannels. b) Cumulative probability histograms for dye loading in $Cx26^{DN}$-expressing HeLa cells. There is no effect of CO_2 (55 mmHg stimulus) but zero Ca^{2+} causes a rightward shift in pixel intensity. c) Median change in median pixel intensity for $Cx26^{WT}$ and $Cx26^{DN}$, showing that the wild type hemichannels responds to CO_2 but $Cx26^{DN}$ does not. Boxes show the interquartile range, the median is indicated by the horizontal line within the box, and the mean is indicated by the cross within the box. Range bars show minimum and maximum values.

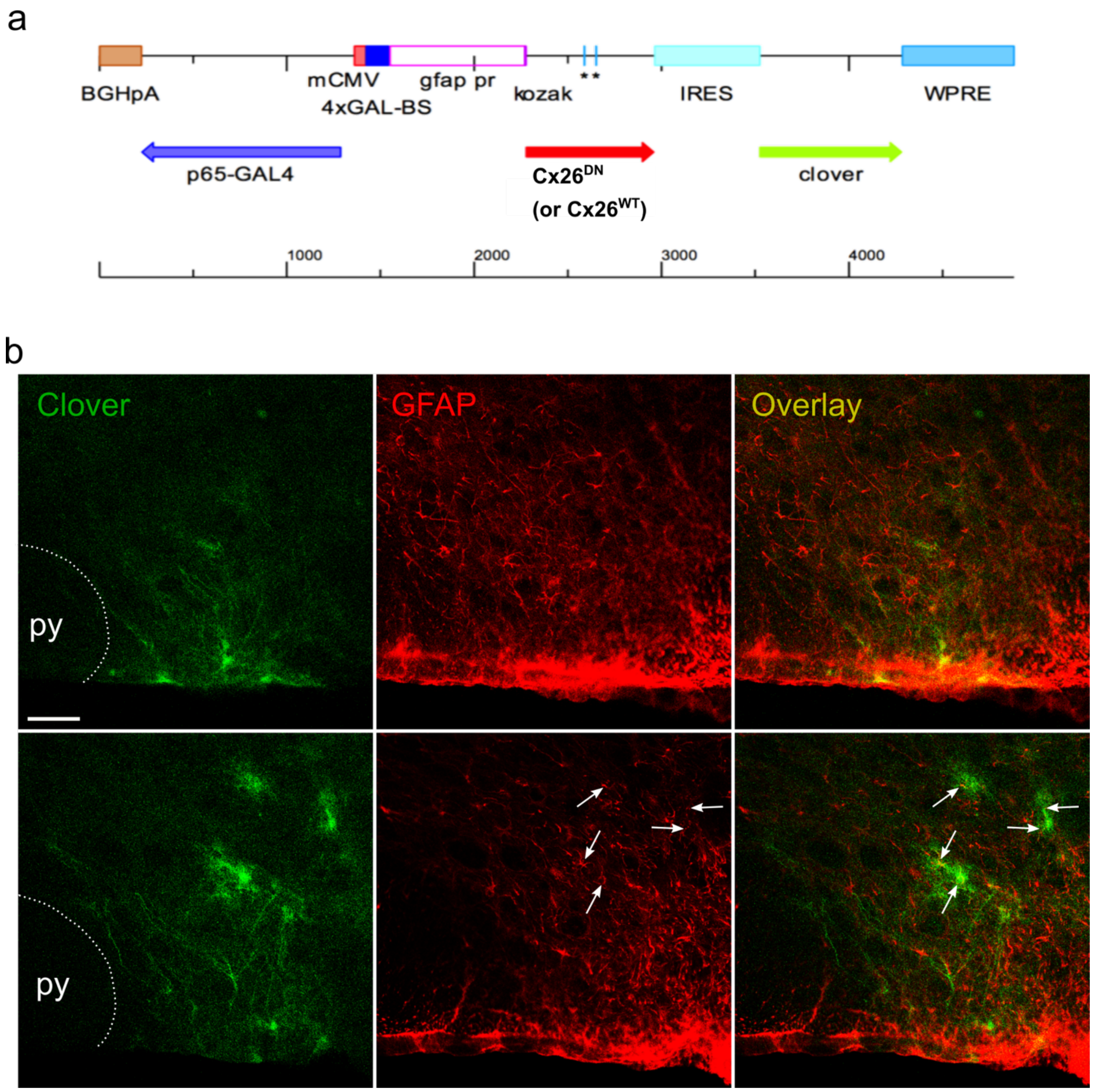

Supplementary Figure 3. a) Lentiviral construct sequences for *in vivo* expression of $Cx26^{WT}$ or $Cx26^{DN}$. b) Demonstration of selective expression of $Cx26^{DN}$ in GFAP+ cells in coronal sections of the caudal parapyramidal area. Montage of Clover fluorescence (green) and GFAP immunoreactivity (red) with overlay. Each image is a single optical section obtained by confocal microscopy. The top row shows ventral cells (within 50 µm of ventral surface). 42/42 Clover labelled cells in this position displayed GFAP immunoreactivity. The bottom row shows more dorsal cells, all of which had typical astrocytic morphology. The white arrows point to puncta of GFAP immunoreactivity present in these cells. Overall 39/40 Clover-labelled cells in the dorsal position had GFAP immunoreactivity. Scale bar 50 µm; py, pyramids.

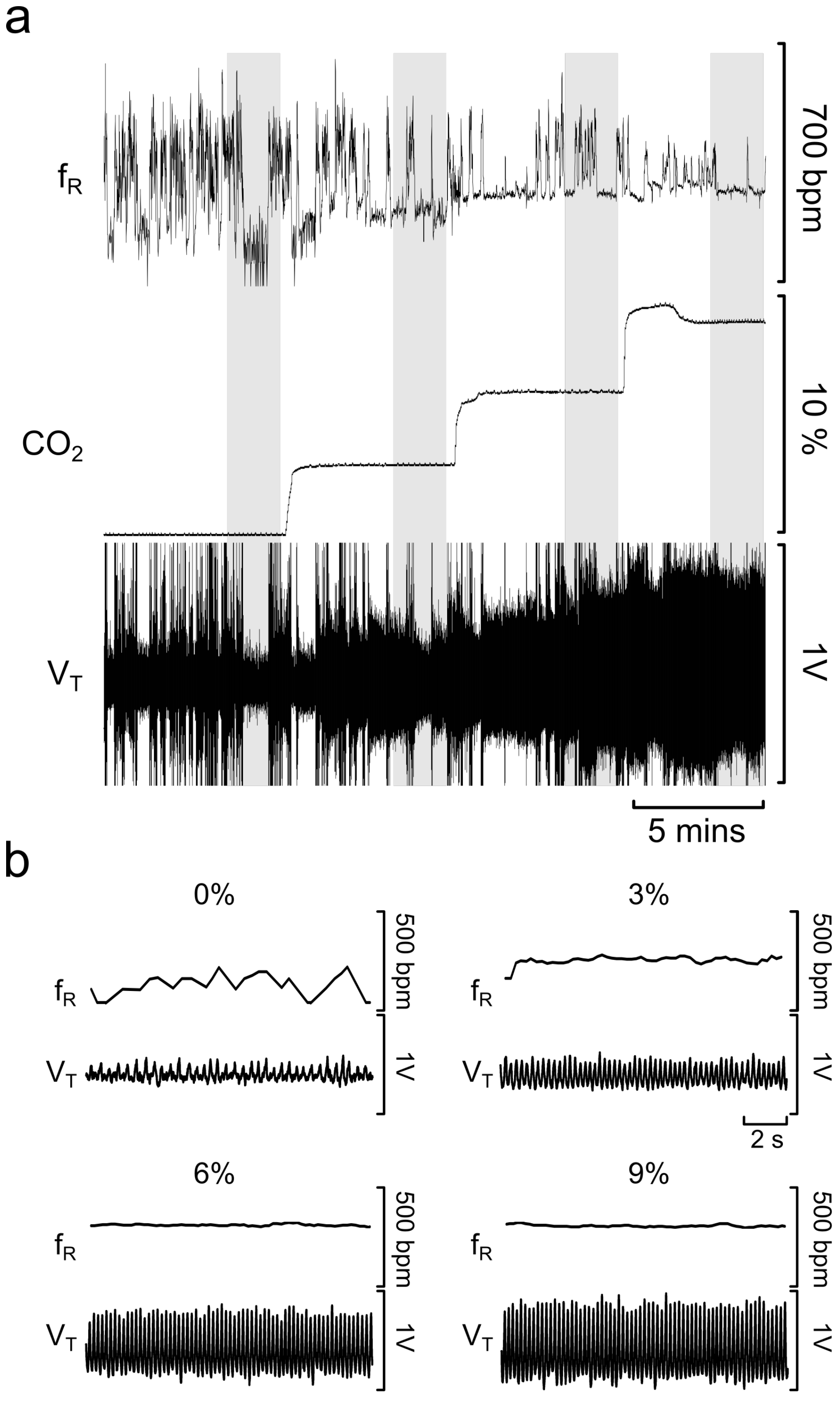

Supplementary Figure 4. Raw plethysmography data. The grey regions show those parts of the experiment selected for analysis of respiratory frequency (f_R) and respiratory flow traces (V_T) for calculation of tidal volume. Large excursions on the V_T trace are movement artefacts.

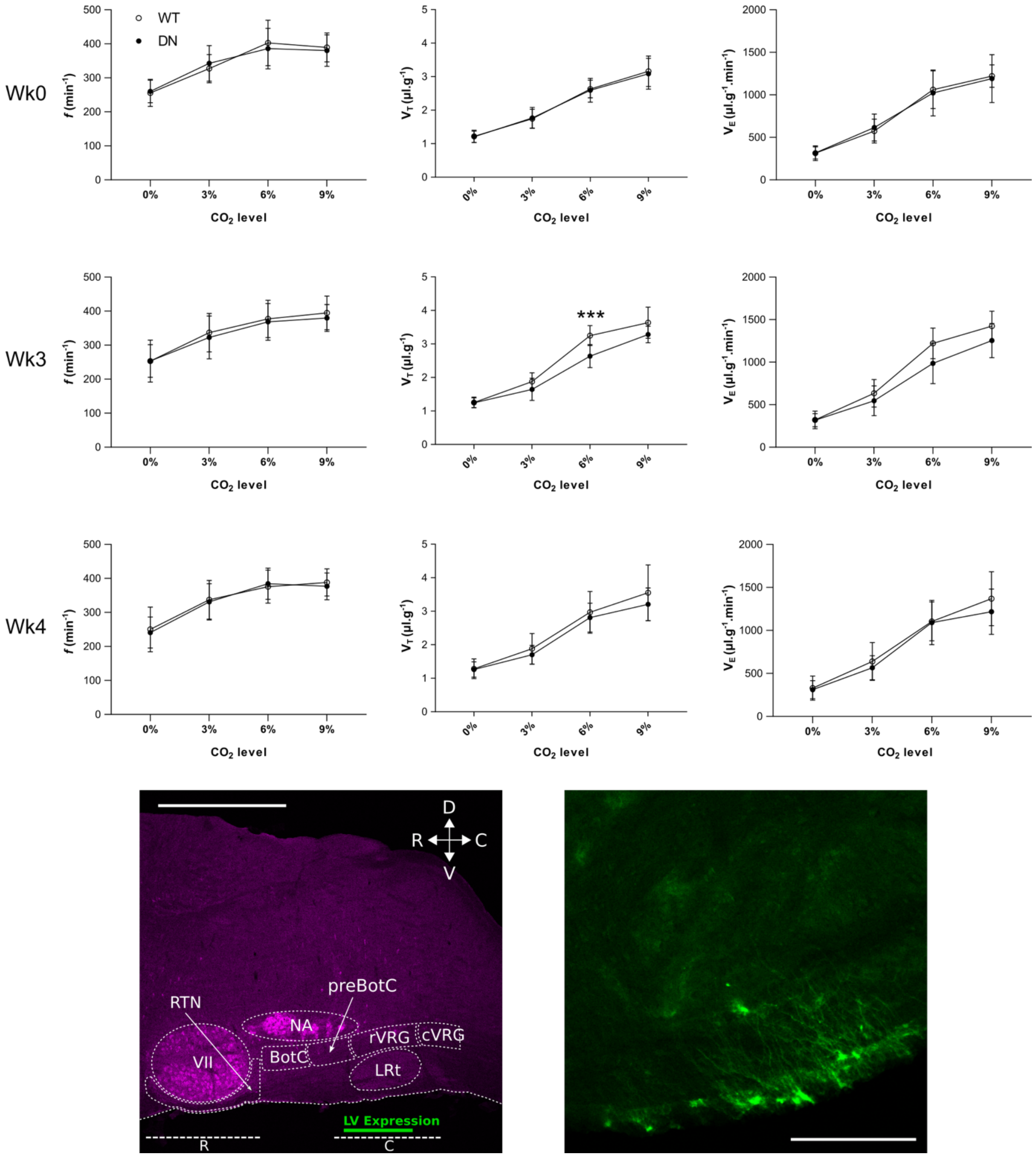

Supplementary Figure 5. Pilot study showing that $Cx26^{DN}$ expression in caudal area near the lateral reticular nucleus (LRt) reduced the tidal volume and minute ventilation responses to 6% CO_2 compared to $Cx26^{WT}$. This effect was apparent at week 3 after viral transduction but had disappeared at week 4. Data plotted as mean ± 95% confidence limits n=6 for $Cx26^{WT}$, n=8 for $Cx26^{DN}$. Scale bars 1mm (left), 200 µm (right). For V_T at 3 weeks the 2-way mixed effect ANOVA was significant p = 0.019 and post-hoc t-tests (one-sided) showed that V_T was reduced at 6% CO_2 (p=0.005, ***); and a marginal effect at 9% CO_2 (p=0.049, which did not pass the false discovery criterion). The 2-way mixed effects ANOVA showed that the differences for minute ventilation (V_E) were not significant (p=0.091).

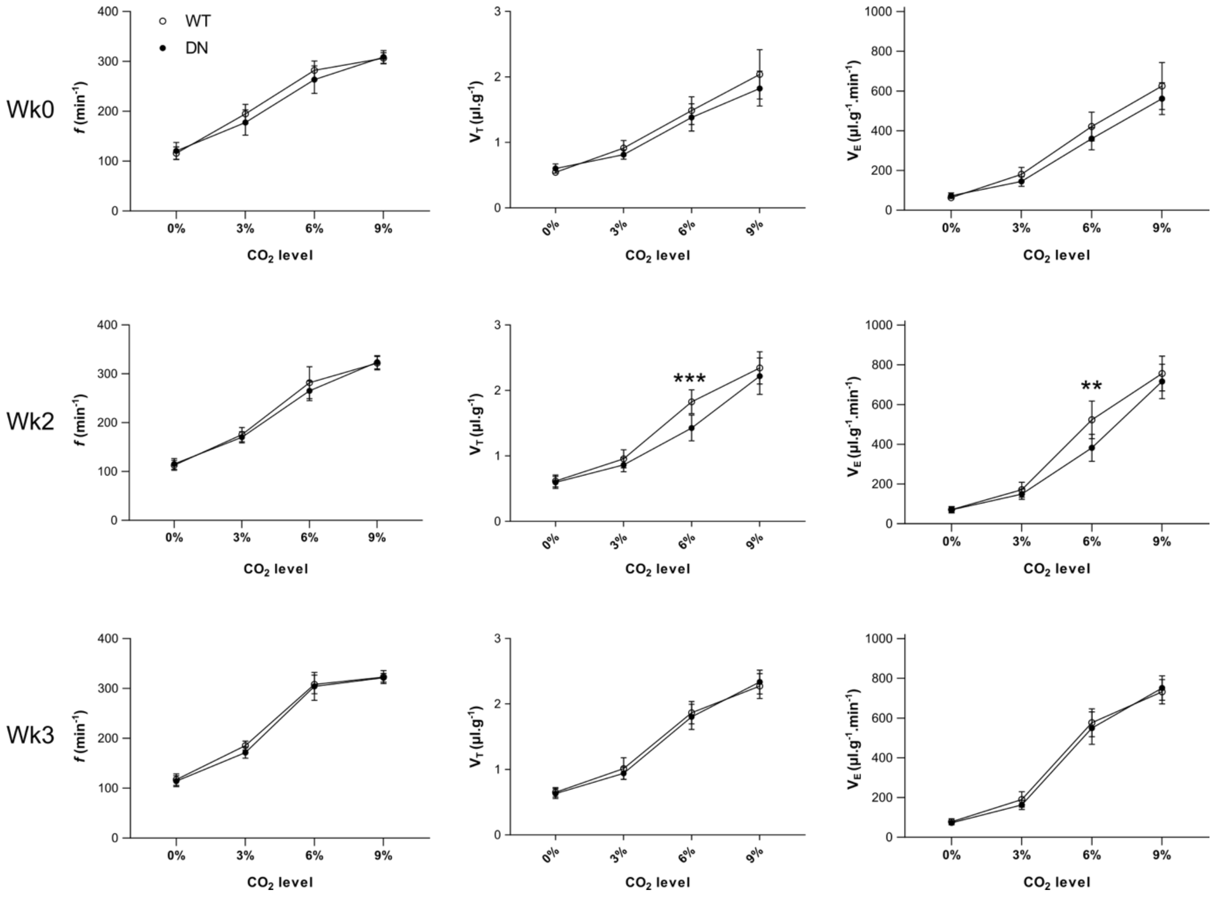

Supplementary Figure 6. Data from Figure 5 of main text plotted as mean ± 95% confidence limits. ***, p=0.002; **, p=0.007, post hoc comparison after 2-way mixed effects ANOVA.

Supplementary Table 1

	Cx26^WT^ ΔV_T (µl.g^{-1})			Cx26^DN^ ΔV_T (µl.g^{-1})		
	3%	6%	9%	3%	6%	9%
**Mean**	**0.34**	**1.21**	**1.73**	**0.27**	**0.83**	**1.62**
SD	0.14	0.24	0.33	0.11	0.26	0.32
**Median**	**0.34**	**1.25**	**1.73**	**0.28**	**0.82**	**1.46**
LQ, UQ	0.29, 0.43	1.05,1.33	1.57, 1.95	0.19, 0.36	0.66, 0.89	1.38, 1.88

% reduction by Cx26^{DN}	3%	**6%**	9%
Means	21	**31**	6
Medians	18	**35**	16

Data from Figure 5, and Supplementary Figure 6 – to quantify the effect on the change in tidal volume (ΔV_T) evoked by 3, 6 and 9% inspired CO_2 of expression of Cx26^{DN} relative to expression of Cx26^{WT} in the caudal parapyramidal area. Cx26^{DN} caused a reduction of ΔV_T of 31% (from means) and 35% (from medians). Although there were reductions ΔV_T at 3 and 9% these were not statistically significant.

Supplementary Table 2

	Cx26^{WT} ΔV_E (µl.g^{-1}.min^{-1})			Cx26^{DN} ΔV_E (µl.g^{-1}.min^{-1})		
	3%	6%	9%	3%	6%	9%
**Mean**	**101**	**452**	**685**	**78**	**312**	**646**
SD	42	143	125	33	100	117
**Median**	**96**	**465**	**690**	**73**	**309**	**648**
LQ, UQ	83,129	404, 551	616, 773	52,108	255, 349	549, 728

% reduction by Cx26^{DN}	3%	**6%**	9%
Means	22	**31**	6
Medians	23	**34**	6

Data from Figure 5, and Supplementary Figure 6 – to quantify the effect on the change in minute ventilation (ΔV_E) evoked by 3, 6 and 9% inspired CO_2 of expression of Cx26^{DN} relative to expression of Cx26^{WT} in the caudal parapyramidal area. Cx26^{DN} caused a reduction of ΔV_E of 31% (from means) and 34% (from medians). Although there were reductions of ΔV_E at 3 and 9% these were not statistically significant.

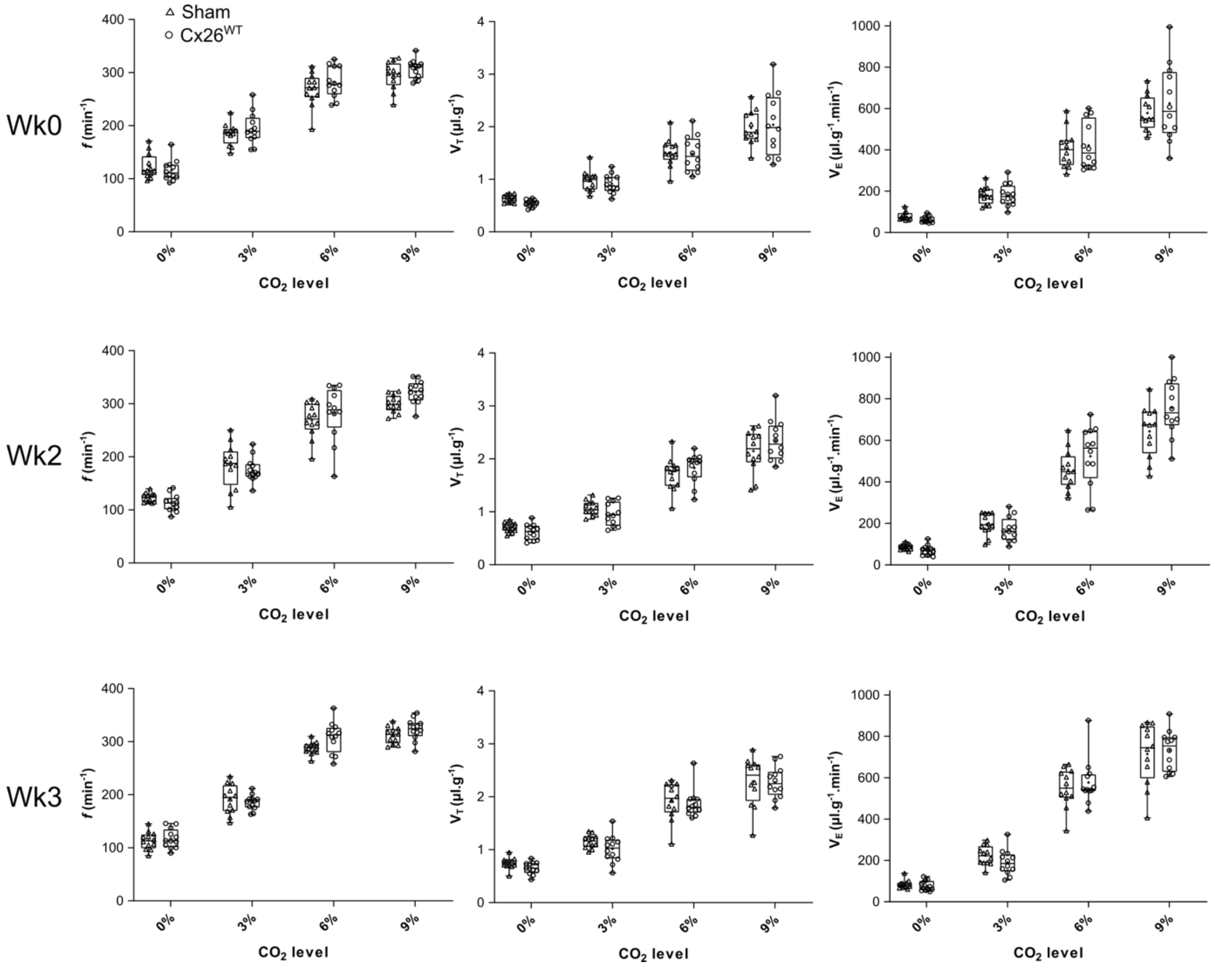

Supplementary Figure 7. Overexpression of Cx26^{WT} does not increase sensitivity to CO_2 compared to sham operated mice. Cx26^{WT} data same as in Figure 5 of main text, the sham operation was performed as part of same study as that of Figure 5. Boxes show the interquartile range, the median is indicated by the horizontal line within the box, and the mean is indicated by the cross within the box. Range bars show minimum and maximum values.

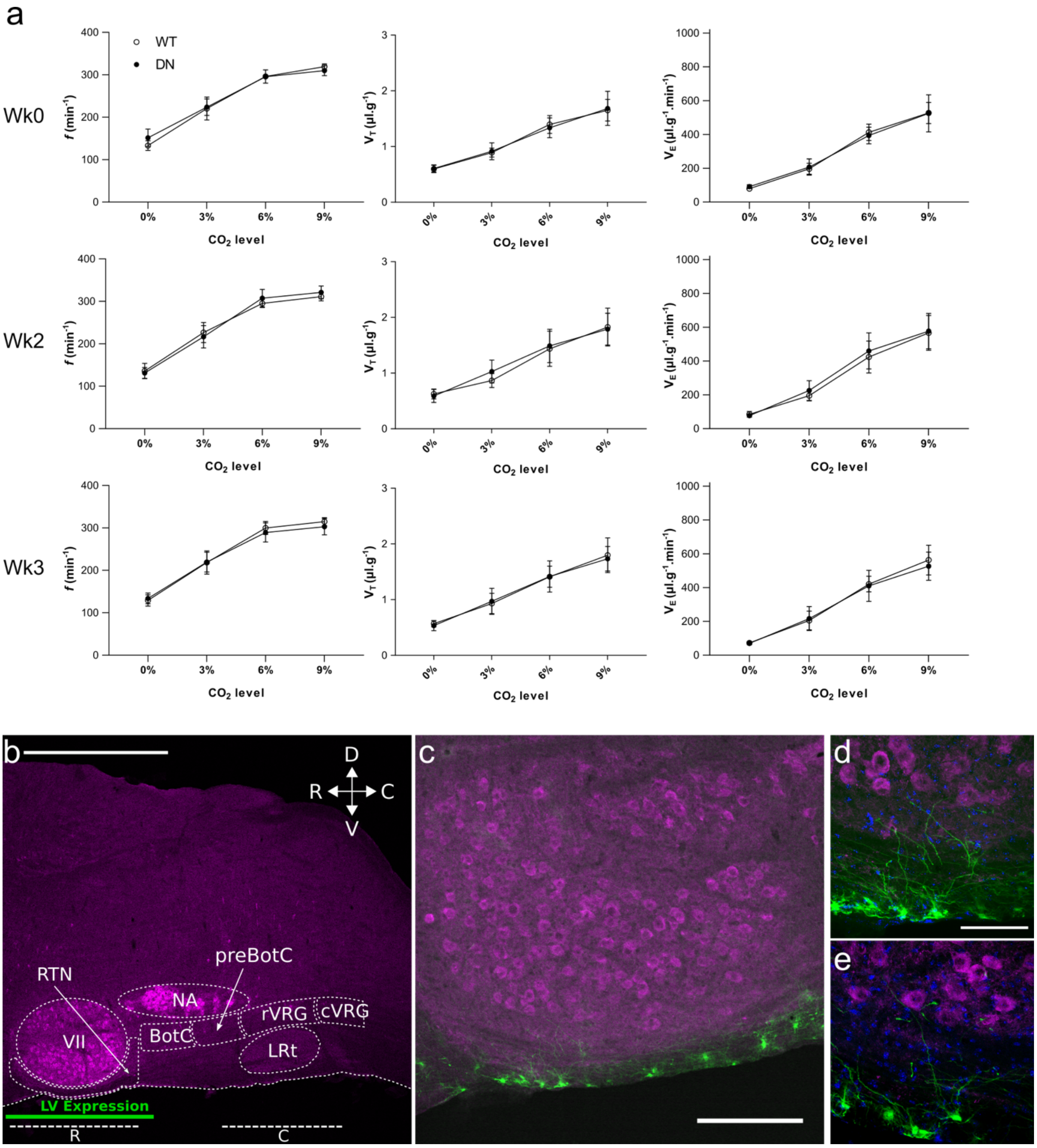

Supplementary Figure 8. a) There is no effect of Cx26^{DN} on the CO_2 sensitivity of beathing when expressed in glial cells of the RTN. Data plotted as mean ± 95% confidence limits. n=8 for both Cx26^{WT} and Cx26^{DN}. b) Location of LV expression. c-e) Micrographs of RTN glial cells transduced. Scale bars: b) 1 mm; c) 200 µm; d,e) 50 µm.

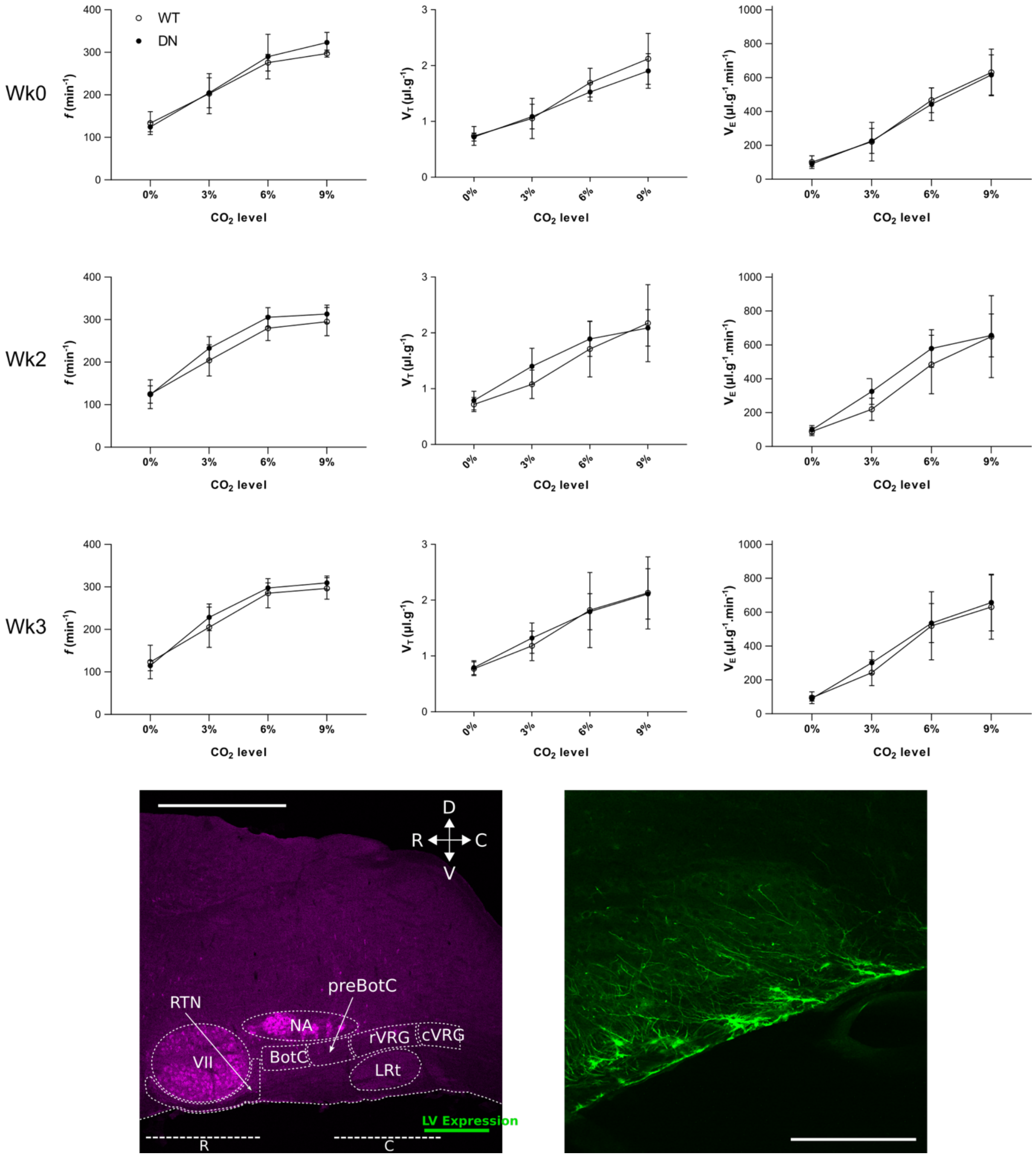

Supplementary Figure 9. Expression of Cx26[DN] in the very caudal medulla oblongata has no effect on the CO_2-sensitivity of breathing. n=6 for Cx26[WT] and Cx26[DN]. Scale bars: left) 1 mm; right) 200 µm.

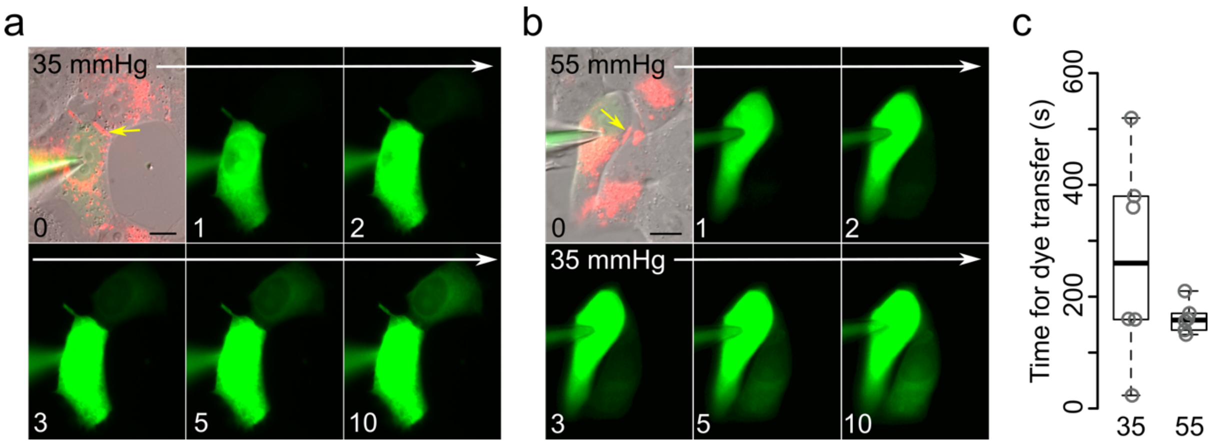

Supplementary Figure 10. Cx26^{DN} forms functional gap junctions that are insensitive to CO_2. Recordings were made with patch electrodes from coupled HeLa cells expressing Cx26^{DN} tagged with mCherry. A patch pipette was used to introduce 2-Deoxy-2-[(7-nitro-2,1,3-benzoxadiazol-4-yl)amino]-D-glucose (NBDG) -a fluorescent glucose analogue -into a single cell (the *donor*) of a coupled pair. The time taken for the dye to diffuse into a coupled cell and achieve 10% of the fluorescence intensity of the donor cell was determined.

a) Montage of images showing superimposed DIC, mCherry (tagged to Cx26^{DN}, red) and NBDG at 0, 1, 2, 3, 5 and 10 minutes after breakthrough at a PCO_2 of 35 mmHg - fluorescence was observed in the coupled cell 3 minutes after breakthrough. Yellow arrows indicate gap junction plaque between cells, scale bar 20 μm.

b) Montage of images showing superimposed DIC, mCherry (tagged to Cx26^{DN}) and NBDG at 0, 1, 2, 3, 5 and 10 minutes after breakthrough starting at a PCO_2 of 55 mmHg, and transferring to 35 mmHg after 2 minutes. The presence of high PCO_2 does not delay entry of NBDG - fluorescence in the coupled cell was observed 3 minutes after breakthrough. Yellow arrows indicate gap junction plaque between cells, scale bar 20 μm.

c) Plot of time taken for fluorescence of coupled cell to reach 10% of that of the donor. The starting PCO_2 makes no difference to the time for permeation of NBDG indicating that the gap junction formed by Cx26^{DN} is insensitive to CO_2 (n=6 pairs of coupled cells for each condition). Boxes show the interquartile range, the median is indicated by the horizontal line within the box. Range bars show minimum and maximum values.
