# Supplementary material for: Connexin26 mediates CO2-dependent regulation of breathing via glial cells of the medulla oblongata
Source: Commun Biol. 2020 Sep 21;3:521. doi: 10.1038/s42003-020-01248-x (PMC7505967; doi:10.1038/s42003-020-01248-x)
Supplement: Supplementary file 2 — Description of Additional Supplementary Files [file 42003_2020_1248_MOESM2_ESM.pdf]

Description of Additional Supplementary Files

File Name: Supplementary Data 1
Description: Source data for the FRET efficiency and Bleaching Efficiency reported in Figure 3.

File Name: Supplementary Data 2
Description: Source data for change in median pixel intensities reported in Figure 4e.

File Name: Supplementary Data 3
Description: Source data for the plethysmography analysis (respiratory frequency, tidal volume and minute ventilation) presented in Figure 5a.
