# Supplementary material for: Connexin26 mediates CO2-dependent regulation of breathing via glial cells of the medulla oblongata
Source: Commun Biol. 2020 Sep 21;3:521. doi: 10.1038/s42003-020-01248-x (PMC7505967; doi:10.1038/s42003-020-01248-x)
Supplement: Supplementary file 6 — Reporting Summary [file 42003_2020_1248_MOESM6_ESM.pdf]

nature research

Corresponding author(s): Nicholas Dale

Last updated by author(s): 05/08/2020

Reporting Summary

Nature Research wishes to improve the reproducibility of the work that we publish. This form provides structure for consistency and transparency in reporting. For further information on Nature Research policies, see our Editorial Policies and the Editorial Policy Checklist.

Statistics

For all statistical analyses, confirm that the following items are present in the figure legend, table legend, main text, or Methods section.

n/a	Confirmed	
☐	☒	The exact sample size (n) for each experimental group/condition, given as a discrete number and unit of measurement
☐	☒	A statement on whether measurements were taken from distinct samples or whether the same sample was measured repeatedly
☐	☒	The statistical test(s) used AND whether they are one- or two-sided<br>*Only common tests should be described solely by name; describe more complex techniques in the Methods section.*
☐	☒	A description of all covariates tested
☐	☒	A description of any assumptions or corrections, such as tests of normality and adjustment for multiple comparisons
☐	☒	A full description of the statistical parameters including central tendency (e.g. means) or other basic estimates (e.g. regression coefficient) AND variation (e.g. standard deviation) or associated estimates of uncertainty (e.g. confidence intervals)
☐	☒	For null hypothesis testing, the test statistic (e.g. F, t, r) with confidence intervals, effect sizes, degrees of freedom and P value noted<br>*Give P values as exact values whenever suitable.*
☒	☐	For Bayesian analysis, information on the choice of priors and Markov chain Monte Carlo settings
☒	☐	For hierarchical and complex designs, identification of the appropriate level for tests and full reporting of outcomes
☒	☐	Estimates of effect sizes (e.g. Cohen's d, Pearson's r), indicating how they were calculated

Our web collection on statistics for biologists contains articles on many of the points above.

Software and code

Policy information about availability of computer code

Data collection	Spike 2 software from CED was used for Plethysmography. Zen Imaging Suite from Zeiss was used for confocal microscopy including FRET. Metafluor software for epifluorescence microscopy
Data analysis	ImageJ for image analysis, Spike 2 software for quantification of plethysmography. SPSS, R, and GraphPAD PRISM for statistical analysis and presentation

For manuscripts utilizing custom algorithms or software that are central to the research but not yet described in published literature, software must be made available to editors and reviewers. We strongly encourage code deposition in a community repository (e.g. GitHub). See the Nature Research guidelines for submitting code & software for further information.

Data

Policy information about availability of data

All manuscripts must include a data availability statement. This statement should provide the following information, where applicable:

- Accession codes, unique identifiers, or web links for publicly available datasets
- A list of figures that have associated raw data
- A description of any restrictions on data availability

All data points are shown in the Figures and the Supplementary Information. Data for the charts in the MS are provided in Supplementary Data 1-3

Field-specific reporting

Please select the one below that is the best fit for your research. If you are not sure, read the appropriate sections before making your selection.

☒ Life sciences ☐ Behavioural & social sciences ☐ Ecological, evolutionary & environmental sciences

For a reference copy of the document with all sections, see nature.com/documents/nr-reporting-summary-flat.pdf

Life sciences study design

All studies must disclose on these points even when the disclosure is negative.

Sample size	For the in vivo experiments a pilot experiment was performed to determine sample size via power calculations. A definitive study was designed according to the power calculation. Details given in text.<br><br>For in vitro (cell culture) experiments no power calculations were performed and sample size was based on previous experiment work in other studies.
Data exclusions	No animals were excluded from statistical analysis. For in vitro experiments all transfections with valid expression were included in the analysis.
Replication	The main finding was effectively replicated 3 times -the pilot experiment, the main experiment (requiring 12 animals/group) which was divided into two halves (6 animals/group) for practical purposes. Although the main experiment was analyzed as a whole (i.e. n=12 per group), the results in the two subgroups replicated the pilot experiment.
Randomization	Mice were randomly allocated into each treatment group
Blinding	Investigators were blinded to group allocation during data acquisition and analysis.

Reporting for specific materials, systems and methods

We require information from authors about some types of materials, experimental systems and methods used in many studies. Here, indicate whether each material, system or method listed is relevant to your study. If you are not sure if a list item applies to your research, read the appropriate section before selecting a response.

Materials & experimental systems

n/a	Involved in the study	
☐	☒	Antibodies
☐	☒	Eukaryotic cell lines
☒	☐	Palaeontology and archaeology
☐	☒	Animals and other organisms
☒	☐	Human research participants
☒	☐	Clinical data
☒	☐	Dual use research of concern

Methods

n/a	Involved in the study	
☒	☐	ChIP-seq
☒	☐	Flow cytometry
☒	☐	MRI-based neuroimaging

Antibodies

Antibodies used	anti-GFAP Abcam, ab68428 or ab4674; anti-ChAT Merck UK, ab144
Validation	These antibodies are widely used and their staining patterns well understood. Staining obtained in this study exactly conformed to these prior expectations.

Eukaryotic cell lines

Policy information about cell lines

Cell line source(s)	HeLa DH from ECACC; HeLa DH cells stably expressing Cx26 from K. Willecke
Authentication	No further authentication other than that from supplier
Mycoplasma contamination	Mycoplasma free
Commonly misidentified lines<br>(See ICLAC register)	*Name any commonly misidentified cell lines used in the study and provide a rationale for their use.*

Animals and other organisms

Policy information about studies involving animals; ARRIVE guidelines recommended for reporting animal research

Laboratory animals	Main experiment: Mice C57BL6. Pilot experiment: mice C57BL6 with floxed Cx26 allele EMMA 00245
Wild animals	None
Field-collected samples	Study did not involve field-collected samples
Ethics oversight	University of Warwick Animal Welfare and Ethical Review Board

Note that full information on the approval of the study protocol must also be provided in the manuscript.
